# Genetic Study of IL6, GDF5 and PAPPA2 in Association with Developmental Dysplasia of the Hip

**DOI:** 10.3390/genes12070986

**Published:** 2021-06-28

**Authors:** Stefan Harsanyi, Radoslav Zamborsky, Lubica Krajciova, Milan Kokavec, Lubos Danisovic

**Affiliations:** 1 Institute of Medical Biology, Genetics and Clinical Genetics, Faculty of Medicine, Comenius University in Bratislava, Sasinkova 4, 811 08 Bratislava, Slovakia; lubica.krajciova@fmed.uniba.sk (L.K.); lubos.danisovic@fmed.uniba.sk (L.D.); 2 Orthopaedic Clinic, Faculty of Medicine, Comenius University in Bratislava and National Institute of Children’s Diseases, Limbova 1, 833 40 Bratislava, Slovakia; radozamborsky@gmail.com (R.Z.); milan.kokavec@nudch.eu (M.K.)

**Keywords:** developmental dysplasia of the hip, DDH, association study, single-nucleotide polymorphism, SNP, genotyping, *IL6*, *GDF5*, *PAPPA2*

## Abstract

Background: Developmental dysplasia of the hip (DDH) is one of the most prevalent skeletal disorders. DDH is considered a pathologic condition with polygenic background, but environmental and mechanic factors significantly contribute to its multifactorial etiology. Inheritance consistent with autosomal dominant type has also been observed. Single-nucleotide polymorphisms (SNPs) in various genes mostly related to formation of connective tissue are studied for a possible association with DDH. Methods: We genotyped three SNPs, rs1800796 located in the promoter region of the *IL6* gene, rs143383 located in the 5′ untranslated region (UTR) of the *GDF5* gene and rs726252 located in the fifth intron of the *PAPPA2* gene. The study consisted of 45 subjects with DDH and 85 controls from all regions of Slovakia. Results: Association between DDH occurrence and studied genotypes affected by aforementioned polymorphisms was confirmed in the case of rs143383 in the *GDF5* gene (*p* = 0.047), where the T allele was over-expressed in the study group. Meanwhile, in the matter of *IL6* and *PAPPA2*, we found no association with DDH (*p* = 0.363 and *p* = 0.478, respectively). Conclusions: These results suggest that there is an association between DDH and *GDF5* polymorphisms and that the T allele is more frequently presents in patients suffering from DDH.

## 1. Introduction

Developmental dysplasia of the hip (DDH) is one the most prevalent skeletal disorders with an estimated incidence ranging from three to six cases per 1000 live births in European countries [1]. It is accompanied by a wide spectrum of anatomical abnormalities of the hip joint such as an incomplete formation of the acetabulum, leading to laxity of the joint capsule, secondary deformity of the proximal femur and irreducible hip dislocation [2]. If the affected individuals are not treated, pain, muscular weakness, growth problems and early osteoarthritis may occur [3].

DDH is considered a pathologic condition with polygenic background, but environmental and mechanic factors do significantly contribute to its multifactorial etiology. The most common risk factors are breech presentation, family history, female gender, swaddling, high birth weight, oligohydramnios and primiparity [4,5]. Neonatal Hip Instability (NHI) or generalized ligamentous hyperlaxity are common occurrences in children and are usually resolved spontaneously in the first eight weeks or six months of age, respectively.

rs1800796 (G > C) is a SNP (single-nucleotide polymorphism) located in the promoter region of the *IL6* (*Interleukin 6*) gene. Its product IL-6 is an immunoregulatory cytokine, which has effects on calcium and vitamin D metabolism, that ultimately affects the metabolism of connective tissue, especially cartilages and bones [6]. Studies conducted in Europe (Croatia) indicated an association of rs1800796 with DDH, but this association was not confirmed in the Chinese Han Population [7,8,9].

rs143383 (C > T) is a SNP located in the 5′ untranslated region (UTR) of the *GDF5* (*Growth/differentiation factor 5*) gene. The GDF5 protein belongs to the TGF-β (Transforming growth factor-β) superfamily and has a role in the regulation of the metabolism of connective tissue, mainly the development of bones and joints and endochondral ossification [10]. A weak association of rs143383 and DDH was reported in the Caucasian population and a more significant association later in a Saudi Arabian population [11,12]. The presence of the risk-associated T allele was excessive in both formerly mentioned studies.

rs726252 (C > T) is a SNP located in the fifth intron of the *PAPPA2* (*Pregnancy-associated plasma protein-A2*) gene. The product is a metalloproteinase (pappalysin 2) with an influence on bone metabolism in mice [13]. The regulation in mice is influenced by the IGF (insulin-like growth factors) signaling pathway [14]. Studies conducted on the Chinese Han Population reported inconsistent results, with one reporting a significant association and the other no association at all [15,16].

The primary goal of the present study was to analyze the association of SNP rs1800796 in the *IL6* gene, rs143383 in the *GDF5* gene and rs726252 in the *PAPPA2* with DDH occurrence in the Caucasian population. A minor goal was to present specific risk factors in the study group that are not related to *IL6*, *GDF5* or *PAPPA2*.

## 2. Materials and Methods

**Subjects:** The study consisted of 45 subjects diagnosed with DDH, 38 females (84.4%) and 7 (15.6) males. The control group contained 85 individuals, of which 51 (60%) were female and 34 (40%) were male. The median age in the study group was 3 years (range 0–49 years) and 15 years in the control group (range 1–27 years). All subjects were examined at the Orthopedic Clinic of the Medical Faculty of Comenius University and the National Institute of Children’s Diseases in Bratislava in the period from 2018 to 2020. To ensure that the majority of the study group was of osteogenic etiology, our method for choosing subjects was based on isolated occurrence of DDH. Because of this, subjects with different etiological backgrounds, e.g., affected by systemic aberrations, different isolated congenital disorders, generalized skeletal dysplasia or rare familial hypermobility syndromes were excluded from the study group. Extensive medical history of subjects and controls was collected. Patients in the control group where chosen based on personal and family history which did not contain any occurrence of DDH or similar familial skeletal defect. Based on USG examination at young age, no individual in the control group exhibited any DDH symptoms. Participants in the study originated from all regions of Slovakia. The study was conducted under the 2013 Declaration of Helsinki and approved by the local ethics committee. Informed consent was obtained from all participants.

**Genotyping:** A total of 130 samples were analysed for studied polymorphisms. Isolation of DNA from peripheral blood was performed using the GeneJET Genomic DNA Purification Kit (Thermo Fisher Scientific, Inc., Waltham, MA, USA), according to the protocols provided by the manufacturer. Three selected SNPs: IL6-rs1800796 (G > C), GDF5-rs143383 (C > T) and PAPPA2-rs726252 (C > T) were genotyped using their respective TaqMan SNP Genotyping Assays on a QuantStudio 3 Real-Time PCR system (Thermo Fisher Scientific, Inc., Waltham, MA, USA). Context sequences in forward orientation IL6: ATG GCC AGG CAG TTC TAC AAC AGC C[C/G]C TCA CAG GGA GAG CCA GAA CAC AGA, GDF5: AAC TCG TTC TTG AAA GGA GAA AGC C[A/G]A CCG CCC CCT TTC TCC TGC ACA ACT and PAPPA2: CTG TAT TAT TCT TCG CTG TCT CTC A[C/T]G CCT TCC TTG TTT CTT GTG AAA AGT. The analyses were performed at the Institute of Medical Biology, Genetics and Clinical Genetics (Faculty of Medicine, Comenius University in Bratislava, Bratislava, Slovakia).

**Statistical analysis:** To compare allele and genotype distributions in the study (DDH) group and controls, Pearson’s chi-square test was used. A legacy chi-square test was used to calculate the departure from Hardy-Weinberg equilibrium (HWE). The distribution of genotypes for *GDF5* and *PAPPA2* in our study was compared to the expected distribution for the Caucasian population reported in Applied Biosystems. The expected distribution for *IL6* was calculated using data from dbSNP, the Database of Single Nucleotide Polymorphisms (https://www.ncbi.nlm.nih.gov/snp/, accessed on 12 April 2021. All statistical analyses were performed with IBM SPSS Statistics version 26 (IBM SPSS Statistics for Windows, Version 26.0, Armonk, NY, USA). The results were considered significant if *p* < 0.05.

## 3. Results

In the study, out of 45 subjects in the DDH group, 10 (22.2%) were initially diagnosed within stage II, 21 (46.7%) within stage III and 14 (31.1%) within stage IV of severity, according to Graf’s ultrasonographic (USG) classification. The affection of left hip joint occurred in 31 (68.9%) cases, right hip affection in 10 (22.2%) cases and four (8.9%) cases were bilateral. 16 (35.5%) subjects had a family history of DDH, three (6.7%) cases had reported connective tissue-related disorders in family history and 26 (57.8%) cases had no DDH-related family history. Comparison of allelic frequencies between our study and international databases is presented in Table 1, as well as the calculation of departure from Hardy-Weinberg equilibrium, which was not statistically significant for any allelic frequency (*p* > 0.05 in all cases). Our observed allelic frequencies are consistent with the expected values for the Caucasian population and to a lesser extent also consistent with expected values for the European population.

To address possible non-*IL6*, *GDF5* or *PAPPA2* related risk factors in the DDH group, 32 (71.1%) women were primiparas and 13 (28.9%) multiparas. 37 (82.2%) subjects came from a physiological pregnancy and eight (17.8%) pregnancies were high-risk. 27 (60%) were positioned head-down and delivered per vias naturales, while 18 (40%) were delivered by cesarean section. Eight (17.8%) subjects were born preterm and 37 (82.2%) were born within the physiological term. At birth, there were five (11.1%) subjects with very low birth weight-VLBW. 38 (84.4%) with normal birth weight-NBW and two (4.4%) with high birth weight-HBW. 10 (22.2%) subjects exhibited short birth length and 35 (77.8%) were in the physiological range. No subjects experienced complications during delivery, nor were they resuscitated upon birth and all exhibited normal psychomotor development.

The distribution of genotypes and alleles in the DDH group and controls are shown in Table 2, Table 3 and Table 4. In Table 2 we present the distribution of genotypes and alleles regarding rs1800796 in *IL6*, where we found no association between the aforementioned polymorphism and distribution of genotypes (*p* = 0.363). The distribution of genotypes affected by rs143383 in *GDF5* is shown in Table 3. In this case, we can see, that the distribution of T alleles and TT genotypes in the DDH group and the control group is significantly different, which is also confirmed by the statistical significance of this association (*p* = 0.047). No statistically significant association has been found between the distribution of alleles and genotypes and rs726252 in *PAPPA2*, presented in Table 4.

## 4. Discussion

DDH is considered a complex multifactorial disorder, with suspicion of autosomal dominant heredity [17]. The majority of genes studied in association with DDH play various roles in the development of connective tissue (ligaments, cartilages and bones), although not all are strictly linked only to DDH. In some cases, polymorphisms in these genes are manifested by a different disorder, where hip dysplasia is not present [18,19]. The polygenic approach suggests that a single polymorphism in an associated gene is not sufficient to cause the disorder, so the creation of a polygenic network could significantly improve the diagnostic and therapeutic approach. In the light of this approach, we need more studies to focus on more SNPs or their combination, which could prove causative for developing DDH [20].

In the present work, we investigated the association of SNPs in *IL6* (rs1800796), *GDF5* (rs143383) and *PAPPA2* (rs726252) genes with the etiopathogenesis of DDH in the Caucasian population. Our study revealed a significant association of one out of three studied polymorphisms, while the other two polymorphisms were found to be DDH non-related in our population. Based on data obtained from patients’ history, we can confirm suspicions of primiparity being a risk factor for DDH, because 71.1% individuals in the study group were firstborns, although due to non-random selection of subjects this result might be affected. We can also confirm, that affection of the left hip joint was more prevalent (68.9%). We did not observe any association with intrauterine position, preterm babies, birth length or birth weight.

The positive association of rs143383 in the *GDF5* gene (*p* = 0.047) was present due to the disequilibrium in the allelic and genomic distributions, where the T alleles and TT genotypes were overrepresented in DDH affected individuals. This polymorphism has first been reported as associated with DDH in Chinese Han females by Dai et al., 2008 [21], however a study by Rouault et al., 2010 [11] reported only a weak association, where instead a different *GDF5* polymorphism (rs143384) was found to be significant [22]. Sadat-Ali et al., 2018 [12] reported a significant association of rs143383 and DDH in a Saudi Arabian population. Baghdadi et al., 2019 reported a disequilibrium in the promoter of *GDF5*, which was hypermethylated in patients with DDH [23]. Polymorphisms in *GDF5* are also studied in association with various types of skeletal dysplasia (e.g., chondrodysplasia or brachydactyly) and osteoarthritis, which is frequently preceded by untreated DDH [24,25].

Studies on rs1800796 in the *IL6* gene were published by Croatian authors, who first looked at the contribution of isolated *IL6* and *TGFB1* polymorphisms to DDH and later on their synergic effect as a risk factor in DDH etiopathogenesis, where the C allele was more frequent in cases than controls [7,8]. The most recent study on *IL6* in the Chinese Han population conducted by Ma et al., 2017 [9] partially supports the claim of previous studies about *TGFB1* and *IL6* association with DDH, although not in the matter of severity, but rather in the onset and development of DDH. Results of our association study indicate that there is no association between rs1800796 in the *IL6* gene and DDH in our population, nor can we confirm the higher prevalence of the C allele, which was scarcely present in both the DDH group and controls. These results are consistent with expected distribution frequencies under HWE for the European/Caucasian population. However, with our present results, we cannot replicate the test for a synergic effect of rs1800796 in *IL6* and rs1800470 in *TGFB1*.

As of yet, there have only been two association studies of *PAPPA2* and DDH in humans, although findings on mice suggest that *PAPPA2* polymorphisms could affect the development of the hip joint by interfering in the metabolism of cartilages via IGF pathway-associated proteins [14]. Both studies of human *PAPPA2* (rs726252) were conducted on the Chinese Han population and came with contradictory results. Dai et al., 2005 [21] suggested that there is a significant association between the *PAPPA2* polymorphism and DDH, especially in comparison of TT genotypes versus other genotypes combined and in females when stratified by gender. Shi et al., 2014 [16] replicated the previous study on larger sample size, but in this case, they found no association between rs726252 and DDH in the Chinese Han population, even if stratified by gender or severity.

Our study presents the first report on a possible association between rs726252 in the *PAPPA2* gene and DDH in the Caucasian population. Although genotype frequency distributions in our population differ from distributions in the Chinese Han population, the outcome of our study is consistent with Shi et al., 2014 [16]. Our results indicate that there is no association between the aforementioned *PAPPA2* polymorphism and DDH in our population; however, when stratified by gender, we observed a marginally significant association between females and DDH. Our findings suggest that, regarding the connection of females to *PAPPA2* polymorphism, further studies on larger sample sizes are necessary to either confirm or refute this.

The major limitations of our study are the small number of DDH affected individuals, along with only a small number of male patients because of the low prevalence of DDH in males. These limitations affected our ability to report a representative stratification by gender or severity. Moreover, even though this study was carried out in patients from multiple regions of Slovakia, not all regions were evenly represented, therefore the distribution of reported alleles and genotypes may have been different. In the future we plan to collect more samples from all regions and test for polymorphisms in more associated genes, hopefully to create a multi-gene comparison chart.

## 5. Conclusions

In conclusion, our study provides the first data obtained by genotyping of DDH affected individuals in Slovakia (Table S1). Based on our results it can be emphasized that, in the Caucasian population, there is a statistically significant association between rs143383 in *GDF5,* and DDH etiopathogenesis. However, in general, the etiology is multifactorial and polygenic, which implies that there are more contributing factors. This association cannot be considered as the only causative element, only a link in a wider network, we strive to understand. Neither *IL6* nor *PAPPA2* share this association. Further studies with different SNPs and a larger number of patients from various regions of our country and the world are needed to widen the base of knowledge of this disorder and the genes affecting its occurrence.

## Figures and Tables

**Table 1:** Comparison of allelic frequencies and departure from HWE between our study and international databases.

Gene	RefSNP	Alleles	Allele Frequencies				
			Our Study (Caucasian)	HapMap (CEU)	1000 Genome (EUR)	Applied Biosystems (Caucasian)	HWE *p*-Value
*IL6*	rs1800796	G/C	0.93/0.07	0.96/0.04	0.95/0.05	-	0.484
*GDF5*	rs143383	C/T	0.40/0.60	0.33/0.67	0.37/0.63	0.39/0.61	0.729
*PAPPA2*	rs726252	C/T	0.45/0.55	0.43/0.57	0.44/0.56	0.43/0.57	0.369

**Table 2:** Distribution of alleles and genotypes in association to rs1800796 in the IL6 gene and DDH occurrence.

(IL6) Group		DDH			Controls			χ2 *p*-Value
Sex		Male n (%)	Female n (%)	All	Male n (%)	Female n (%)	All	
Allele Σ		14	76	90	68	102	170	-
Allele	G	12 (13.4)	71 (78.9)	83	64 (40.0)	96 (60.0)	160	
	C	2 (2.2)	5 (5.5)	7	4 (40.0)	6 (60.0)	10	
Genotype Σ		7	38	45	34	51	85	0.363
Genotype	GG	6 (13.3)	33 (73.3)	39	32 (37.6)	45 (52.9)	77	
	GC	- (0)	5 (11.1)	5	2 (2.4)	6 (7.1)	8	
	CC	1 (2.3)	- (0)	1	- (0)	- (0)	0	

**Table 3:** Distribution of alleles and genotypes in association to rs143383 in the GDF5 gene and DDH occurrence.

(GDF5) Group		DDH			Controls			χ2 *p*-Value
Sex		Male n (%)	Female n (%)	All	Male n (%)	Female n (%)	All	
Allele Σ		14	76	90	68	102	170	-
Allele	C	6 (6.7)	22 (24.4)	28	35 (20.6)	41 (24.1)	76	
	T	8 (8.9)	54 (60.0)	62	33 (19.4)	61 (35.9)	94	
Genotype Σ		7	38	45	34	51	85	0.047
Genotype	CC	1 (2.3)	2 (4.4)	3	11 (12.9)	7 (8.2)	18	
	CT	4 (8.9)	18 (40.0)	22	14 (16.5)	29 (34.1)	43	
	TT	2 (4.4)	18 (40.0)	20	9 (10.6)	15 (17.6)	24	

**Table 4:** Distribution of alleles and genotypes in association to rs726252 in the PAPPA2 gene and DDH occurrence.

(PAPPA2) Group		DDH			Controls			χ2 *p*-Value
Sex		Male n (%)	Female n (%)	All	Male n (%)	Female n (%)	All	
Allele Σ		14	76	90	68	102	170	-
Allele	C	9 (10.0)	27 (30.0)	36	26 (15.3)	56 (32.9)	82	
	T	5 (5.5)	49 (54.5)	54	42 (24.7)	46 (27.1)	88	
Genotype Σ		7	38	45	34	51	85	0.478
Genotype	CC	3 (6.6)	5 (11.1)	8	5 (5.9)	17 (20.0)	22	
	CT	3 (6.6)	17 (37.8)	20	16 (18.8)	22 (25.9)	38	
	TT	1 (2.3)	16 (35.6)	17	13 (15.4)	12 (14.1)	25	

## Data Availability

The following are available online at: https://www.mdpi.com/article/10.3390/genes12070986/s1. Supplementary database: Subject database including studied risk factors, clinicopathological variables a results of genotyping.

## Supplementary Materials

The following are available online at https://www.mdpi.com/article/10.3390/genes12070986/s1, Table S1: Subject database including studied risk factors, clinicopathological variables a results of geno-typing.
