# Supplementary material for: Genetic Study of IL6, GDF5 and PAPPA2 in Association with Developmental Dysplasia of the Hip
Source: Genes (Basel). 2021 Jun 28;12(7):986. doi: 10.3390/genes12070986 (PMC8303839; doi:10.3390/genes12070986)
Supplement: Supplementary file 1 [file genes-12-00986-s001.zip › genes-1213286-supplementary.pdf]

Table S1: Subject database including studied risk factors, clinicopathological variables a results of geno-typing

| No. | Sex | Family hist. | Gravi | Pregnancy | Gest. wk | Delivery | BW(g) | BL(cm) | Hip | USG exam. | Occurence | IL6 | GDF5 | PAPPA2 |
|---|---|---|---|---|---|---|---|---|---|---|---|---|---|---|
| 1 | F | gm osteopor | 2 | physiological | 38 | sc | 2610 | 49 | left | IV | DDH | GG | CT | CC |
| 2 | F | gf ddh | 1 | physiological | term | head-down | 3600 | 50 | right | III | DDH | GG | CT | CT |
| 3 | F | no | 3 | physiological | 41 | head-down | 3700 | 54 | left | II | DDH | GG | CT | TT |
| 4 | F | no | 3 | high-risk | 42 | sc | 4017 | 49 | right | III | DDH | GG | CT | TT |
| 5 | M | gm skleroder | 1 | physiological | 37 | head-down | 2320 | 46 | left | II | DDH | GG | CT | CT |
| 6 | F | m harness | 1 | physiological | 38 | head-down | 2550 | 44 | left | IV | DDH | GC | CT | CT |
| 7 | F | no | 1 | physiological | 39 | head-down | 3390 | 49 | left | III | DDH | GG | CT | TT |
| 8 | F | no | 1 | gestational diabetes | term | head-down | 3230 | 51 | left | III | DDH | GG | TT | CT |
| 9 | F | no | 1 | physiological | 38 | head-down | 3470 | 50 | left | IV | DDH | GG | TT | TT |
| 10 | F | m ddh | 1 | high-risk | 40 | breech - sc | 3020 | 49 | right | III | DDH | GG | TT | TT |
| 11 | F | m harness | 2 | high-risk | 39 | sc | 3120 | 47 | bilateral | IV | DDH | GG | TT | CT |
| 12 | F | no | 1 | physiological | 39 | sc | 2750 | 48 | left | IV | DDH | GG | TT | TT |
| 13 | F | no | 2 | physiological | 39 | sc | 3140 | 50 | left | II | DDH | GC | TT | CT |
| 14 | F | no | 1 | physiological | 39 | sc | 2930 | 47 | left | IV | DDH | GG | CT | TT |
| 15 | F | no | 1 | physiological | 39 | sc | 3710 | 50 | left | II | DDH | GG | CT | CT |
| 16 | F | m harness | 2 | physiological | term | head-down | normal | 48 | left | II | DDH | GG | TT | TT |
| 17 | M | no | 1 | physiological | term | sc | normal | 50 | left | II | DDH | GG | CT | CC |
| 18 | F | gm, m, f | 1 | physiological | term | head-down | 2700 | 48 | left | III | DDH | GG | TT | CT |
| 19 | F | gm coxarthro | 3 | physiological | 37 | sc | 3010 | 49 | left | III | DDH | GG | CT | CC |
| 20 | F | no | 1 | physiological | 35 | head-down | 2050 | 46 | left | III | DDH | GG | TT | CT |
| 21 | M | -sister, gm dd | 1 | physiological | 41 | head-down | 4300 | 52 | right | III | DDH | GG | CT | CC |
| 22 | F | gm dhh, f-siste | 1 | physiological | 39 | breech - sc | 2450 | 47 | left | III | DDH | GG | CT | CT |
| 23 | M | no | 2 | physiological | 41 | head-down | 3350 | 50 | left | III | DDH | GG | CT | CT |
| 24 | F | harness, f-sist | 2 | physiological | 38 | head-down | 3480 | 50 | left | III | DDH | GG | CT | CT |
| 25 | F | no | 1 | physiological | 41 | head-down | 4400 | 53 | left | III | DDH | GG | TT | CT |
| 26 | M | sister healthy gemini | | high-risk | 36 | sc | 2500 | 47 | left | IV | DDH | GG | CC | CT |
| 27 | F | no | 3 | physiological | term | head-down | 2900 | 50 | right | IV | DDH | GG | TT | TT |
| 28 | M | yes | 1 | physiological | term | head-down | 3000 | 50 | left | III | DDH | GG | TT | CC |
| 29 | F | no | 1 | physiological | term | head-down | 3750 | 51 | left | II | DDH | GC | CT | TT |
| 30 | F | m harness | 1 | physiological | term | head-down | 3300 | 50 | left | IV | DDH | GG | CT | CT |

| | | | | | | | | | | | | | | |
|---|---|---|---|---|---|---|---|---|---|---|---|---|---|---|
| 31 | F | no | 1 | physiological | term | head-down | 3000 | 50 | left | II | DDH | GG | TT | TT |
| 32 | F | no | 1 | physiological | 36 | breech - sc | 2660 | 48 | left | III | DDH | GC | CT | CT |
| 33 | F | no | 1 | physiological | term | sc | 3870 | 50 | left | III | DDH | GG | TT | CT |
| 34 | F | m harness | 1 | physiological | 42 | head-down | 3690 | 50 | left | III | DDH | GG | CC | CC |
| 35 | F | no | 1 | high-risk | 37 | head-down | 2320 | 44 | right | III | DDH | GG | TT | TT |
| 36 | F | no | 2 | physiological | 39 | head-down | 3910 | 53 | right | IV | DDH | GG | CT | CT |
| 37 | F | gf ddh | 1 | physiological | term | head-down | 3520 | 51 | right | IV | DDH | GG | TT | CT |
| 38 | F | f ddh | 1 | physiological | term | head-down | normal | 50 | right | II | DDH | GG | TT | CT |
| 39 | F | gm, gf | 1 | physiological | term | head-down | 3750 | 49 | bilateral | IV | DDH | GG | CT | TT |
| 40 | F | no | 1 | physiological | term | sc | 3360 | 52 | bilateral | IV | DDH | GG | CC | TT |
| 41 | F | gf-brother | 1 | physiological | 35 | sc | 2320 | 45 | right | IV | DDH | GG | TT | CC |
| 42 | F | no | 1 | high-risk | 39 | breech - sc | 3300 | 48 | left | III | DDH | GC | TT | TT |
| 43 | F | no | 5 | physiological | term | head-down | 2840 | 49 | bilateral | III | DDH | GG | CT | CC |
| 44 | F | no | 3 | high-risk | 32 | sc | 1960 | 45 | left | III | DDH | GG | CT | TT |
| 45 | M | no | 1 | physiological | term | head-down | normal | 50 | left | II | DDH | CC | TT | TT |
| 46 | M | no | 3 | physiological | term | head-down | 4280 | 54 | healthy | I | Control | GG | CC | TT |
| 47 | M | no | 1 | physiological | term | head-down | 3300 | 49 | healthy | I | Control | GG | CT | CT |
| 48 | M | no | 3 | physiological | term | head-down | 3800 | 50 | healthy | I | Control | GG | TT | CT |
| 49 | F | no | 1 | high-risk | term | head-down | 2400 | 48 | healthy | I | Control | GG | CC | TT |
| 50 | F | no | 3 | high-risk | 32 | sc | 1750 | 40 | healthy | I | Control | GG | CT | CT |
| 51 | F | no | 1 | physiological | term | sc | 4450 | 54 | healthy | I | Control | GG | TT | CC |
| 52 | M | no | 2 | physiological | term | head-down | 3500 | 50 | healthy | I | Control | GG | TT | TT |
| 53 | M | no | 2 | physiological | 37 | head-down | 2950 | 51 | healthy | I | Control | GG | TT | CT |
| 54 | M | no | 4 | physiological | 40 | head-down | 3200 | 50 | healthy | I | Control | GG | TT | CT |
| 55 | F | no | 2 | physiological | term | sc | 3500 | 53 | healthy | I | Control | GC | CC | CT |
| 56 | F | no | 1 | physiological | term | head-down | 3400 | 50 | healthy | I | Control | GG | CC | TT |
| 57 | M | no | 3 | physiological | term | head-down | 4125 | 51 | healthy | I | Control | GG | CC | CC |
| 58 | M | no | 2 | physiological | term | head-down | 3520 | 49 | healthy | I | Control | GG | CC | CT |
| 59 | F | no | 2 | physiological | 33 | head-down | 3170 | 50 | healthy | I | Control | GG | CT | CT |
| 60 | M | no | 2 | physiological | term | sc | 2920 | 52 | healthy | I | Control | GG | CT | CT |
| 61 | M | no | 1 | physiological | 41 | head-down | 3800 | 50 | healthy | I | Control | GG | CC | CT |

| | | | | | | | | | | | | | | |
|---|---|---|---|---|---|---|---|---|---|---|---|---|---|---|
| 62 | F | no | 2 | physiological | 38 | sc | 3480 | 50 | healthy | I | Control | GG | CT | CT |
| 63 | M | no | 4 | physiological | term | head-down | 3500 | 50 | healthy | I | Control | GG | CC | CC |
| 64 | M | no | 2 | high-risk | 42 | head-down | 3280 | 50 | healthy | I | Control | GG | TT | CT |
| 65 | M | no | 3 | physiological | 38 | head-down | 2890 | 49 | healthy | I | Control | GG | CT | CT |
| 66 | F | no | 4 | physiological | term | breech | 3010 | 51 | healthy | I | Control | GG | CC | CT |
| 67 | F | no | 1 | high-risk | 39 | sc | 3100 | 50 | healthy | I | Control | GG | TT | CT |
| 68 | F | no | 1 | physiological | term | head-down | 3600 | 50 | healthy | I | Control | GG | CT | CT |
| 69 | F | no | 2 | physiological | 40 | head-down | 3000 | 49 | healthy | I | Control | GG | CT | CC |
| 70 | F | no | 2 | physiological | term | head-down | 4350 | 52 | healthy | I | Control | GG | CT | CT |
| 71 | F | no | 2 | physiological | 40 | head-down | 3250 | 51 | healthy | I | Control | GG | CC | CT |
| 72 | M | no | 1 | physiological | 41 | sc | 3550 | 51 | healthy | I | Control | GG | CC | CT |
| 73 | F | no | 1 | physiological | 39 | head-down | 3050 | 50 | healthy | I | Control | GG | CT | TT |
| 74 | M | no | 1 | physiological | term | head-down | 3980 | 52 | healthy | I | Control | GG | CT | CT |
| 75 | F | no | 1 | physiological | term | head-down | 3000 | 51 | healthy | I | Control | GG | TT | CC |
| 76 | F | no | 2 | physiological | 34 | sc | 2350 | 46 | healthy | I | Control | GG | CT | CC |
| 77 | F | no | 1 | physiological | term | head-down | 2950 | 50 | healthy | I | Control | GG | CT | CT |
| 78 | F | no | 1 | physiological | term | head-down | 3100 | 54 | healthy | I | Control | GG | TT | TT |
| 79 | F | no | 1 | physiological | term | head-down | 3300 | 51 | healthy | I | Control | GG | TT | CT |
| 80 | F | no | 3 | physiological | term | head-down | 3200 | 48 | healthy | I | Control | GG | CT | TT |
| 81 | F | no | 1 | physiological | term | head-down | 2600 | 54 | healthy | I | Control | GG | CT | CC |
| 82 | M | no | 4 | high-risk | 38 | sc | 3400 | 50 | healthy | I | Control | GC | CT | CT |
| 83 | M | no | 2 | physiological | term | head-down | 2690 | 53 | healthy | I | Control | GG | TT | CT |
| 84 | M | no | 2 | physiological | term | sc | 3950 | 43 | healthy | I | Control | GG | CT | CT |
| 85 | F | no | 3 | physiological | term | head-down | 3200 | 49 | healthy | I | Control | GG | CC | TT |
| 86 | M | no | 1 | physiological | term | sc | 3500 | 50 | healthy | I | Control | GG | CT | CT |
| 87 | F | no | 2 | physiological | 36 | breech | 2650 | 46 | healthy | I | Control | GG | CT | CT |
| 88 | F | no | 3 | physiological | term | head-down | 3500 | 43 | healthy | I | Control | GC | CT | CC |
| 89 | F | no | 2 | physiological | term | head-down | 3120 | 49 | healthy | I | Control | GG | TT | TT |
| 90 | F | no | 1 | physiological | term | head-down | 3500 | 51 | healthy | I | Control | GC | CT | CT |
| 91 | M | no | 1 | physiological | post-term | head-down | 3000 | 50 | healthy | I | Control | GG | CT | TT |
| 92 | F | no | 2 | gestational diabetes | 37 | head-down | 3310 | 48 | healthy | I | Control | GC | CT | CT |

| | | | | | | | | | | | | | | |
|---|---|---|---|---|---|---|---|---|---|---|---|---|---|---|
| 93 | M | no | 1 | physiological | term | sc | 3880 | 52 | healthy | I | Control | GG | CC | TT |
| 94 | F | no | 2 | physiological | term | head-down | 2900 | 51 | healthy | I | Control | GG | CT | CT |
| 95 | F | no | 1 | physiological | term | head-down | 4550 | 55 | healthy | I | Control | GG | TT | TT |
| 96 | M | no | 2 | high-risk | term | head-down | 3950 | 52 | healthy | I | Control | GG | CT | TT |
| 97 | M | no | 1 | preeklampsia | 38 | head-down | 2000 | 47 | healthy | I | Control | GG | TT | CT |
| 98 | F | no | 1 | physiological | term | head-down | normal | 50 | healthy | I | Control | GG | CT | TT |
| 99 | M | no | 7 | physiological | 36 | head-down | 2280 | 45 | healthy | I | Control | GG | TT | CC |
| 100 | M | no | 2 | physiological | term | head-down | 3650 | 51 | healthy | I | Control | GG | CT | CC |
| 101 | F | no | 2 | physiological | term | head-down | 3500 | 51 | healthy | I | Control | GG | CT | CT |
| 102 | F | no | 2 | physiological | term | sc | 3800 | 53 | healthy | I | Control | GG | CT | CT |
| 103 | F | no | 1 | physiological | term | sc | 2700 | 46 | healthy | I | Control | GG | CT | CC |
| 104 | F | no | 2 | physiological | term | head-down | 4000 | 52 | healthy | I | Control | GG | TT | CC |
| 105 | M | no | 2 | physiological | term | head-down | 3500 | 50 | healthy | I | Control | GG | CC | TT |
| 106 | M | no | 1 | physiological | 40 | head-down | 3100 | 52 | healthy | I | Control | GG | CC | TT |
| 107 | F | no | 1 | physiological | term | sc | 3500 | 55 | healthy | I | Control | GG | TT | CT |
| 108 | F | no | 2 | high-risk | term | sc | 3290 | 50 | healthy | I | Control | GG | TT | CT |
| 109 | F | no | 3 | physiological | term | head-down | 3400 | 51 | healthy | I | Control | GG | TT | CC |
| 110 | F | no | 2 | physiological | 37 | sc | 2900 | 51 | healthy | I | Control | GG | CT | CC |
| 111 | F | no | 3 | physiological | 38 | sc | 2800 | 47 | healthy | I | Control | GG | CT | CC |
| 112 | F | no | 3 | physiological | term | head-down | 3430 | 53 | healthy | I | Control | GG | TT | TT |
| 113 | F | no | 1 | physiological | term | head-down | 3180 | 52 | healthy | I | Control | GG | CT | CT |
| 114 | F | no | 3 | physiological | term | head-down | 3700 | 49 | healthy | I | Control | GG | TT | CC |
| 115 | F | no | 1 | physiological | term | sc | 3510 | 51 | healthy | I | Control | GG | CT | CT |
| 116 | F | no | 1 | physiological | term | sc | 4200 | 53 | healthy | I | Control | GC | TT | TT |
| 117 | M | no | 2 | physiological | 37 | head-down | 3510 | 51 | healthy | I | Control | GG | CT | TT |
| 118 | F | no | 2 | physiological | 40 | head-down | 3500 | 51 | healthy | I | Control | GG | CT | CT |
| 119 | F | no | 4 | physiological | term | head-down | 3200 | 49 | healthy | I | Control | GG | CT | CC |
| 120 | M | no | 3 | physiological | 38 | sc | 3800 | 51 | healthy | I | Control | GG | CC | TT |
| 121 | M | no | 2 | physiological | term | head-down | 3750 | 51 | healthy | I | Control | GG | CT | TT |
| 122 | M | no | 1 | high-risk | term | head-down | 3000 | 50 | healthy | I | Control | GG | CT | TT |
| 123 | F | no | 1 | physiological | term | head-down | 3100 | 51 | healthy | I | Control | GC | CT | TT |

| | | | | | | | | | | | | | | |
|---|---|---|---|---|---|---|---|---|---|---|---|---|---|---|
| 124 | F | no | 1 | physiological | term | head-down | 2810 | 47 | healthy | I | Control | GG | CT | CC |
| 125 | F | no | 1 | physiological | term | head-down | 2800 | 49 | healthy | I | Control | GG | CC | CC |
| 126 | F | no | 1 | physiological | term | head-down | 3720 | 52 | healthy | I | Control | GG | CT | CC |
| 127 | F | no | 1 | physiological | 38 | sc | 3700 | 50 | healthy | I | Control | GG | TT | CC |
| 128 | M | no | 4 | physiological | 40 | head-down | 4100 | 54 | healthy | I | Control | GG | CT | TT |
| 129 | M | no | 1 | physiological | 38 | sc | 3750 | 49 | healthy | I | Control | GC | TT | TT |
| 130 | M | no | 1 | physiological | term | head-down | normal | 50 | healthy | I | Control | GG | CC | CC |
